# Renal osteodystrophy in a sample of patients on dialysis in Northeastern Brazil: a cross-sectional analysis

**DOI:** 10.1590/2175-8239-JBN-2024-0174en

**Published:** 2025-04-14

**Authors:** Luiz Alberto Soares de Araújo Coutinho, Ana Paula Santana Gueiros, Júlia Braga Vaz, Eduarda Cerqueira Russo, Vanda Jorgetti, José Edevanilson de Barros Gueiros

**Affiliations:** 1 Universidade Federal de Pernambuco, Empresa Brasileira de Serviços Hospitalares, Hospital das Clínicas, Serviço de Nefrologia, Recife, PE, Brazil.; 2 Universidade de São Paulo, Faculdade de Medicina, Laboratório de Investigação Médica, São Paulo, SP, Brazil.

**Keywords:** Renal Insufficiency, Chronic, Dialysis, Chronic Kidney Disease-Mineral and Bone Disorder, Bone Biopsy

## Abstract

**Introduction:**

Mineral and bone meta-bolism disorders in chronic kidney disease (CKD-MBD) are one of the most significant complications of CKD. The objective of this study was to describe the types of renal osteodystrophy (ROD) and the clinical and osteometabolic profiles of dialysis patients undergoing bone biopsy in Pernambuco.

**Methods:**

A cross-sectional, retrospective study that assessed patients biopsied between January 2004 and September 2021 was conducted. Patients receiving conservative treatment for CKD, kidney transplant recipients, and those with inadequate biopsies were excluded. All clinical and laboratory parameters were from the time of biopsy.

**Results:**

We assessed 250 patients with median age of 48 years (57.6% women) and on dialysis for a median time of 108 months. Regarding the diagnosis of ROD, the prevalence was: osteitis fibrosa (54.5%), mixed disease (30.4%), adynamic bone disease (12.4%), and osteomalacia (3.2%). The prevalences of osteoporosis, aluminum toxicity, and iron toxicity were 43.6%, 46.8% and 27.5%, respectively.

**Conclusions:**

We demonstrated a high prevalence of diseases related to high bone turnover and aluminum toxicity.

## Introduction

Mineral and bone metabolism disorders in chronic kidney disease (CKD-MBD) are one of the most significant complications of CKD^1^. As a consequence of CKD-MBD, dialysis patients suffer from bone loss and have an increased risk of fractures, in addition to presenting early and accelerated vascular calcification. These manifestations add up and contribute to poor clinical outcomes and high cardiovascular mortality in these patients^1,2,3^.

The term renal osteodystrophy (ROD) is used to describe histological changes assessed through semiquantitative and histomorphometric analyses of bone biopsy, using the TMV system (turnover, mineralization, and volume). ROD is therefore one of the components of CKD-MBD and may be classified as high or low bone turnover, whereby high turnover comprises diagnoses of osteitis fibrosa (OF) and mixed disease (MD), while low turnover is represented by adynamic bone disease (ABD) and osteomalacia (OM). Aluminum toxicity, at different degrees, may be present in any type of ROD^4,5^.

In Brazil, there are few centers specialized in the treatment of CKD-MBD. Performing bone biopsy procedures and, more specifically, the processing and diagnostic analysis of the samples are even more restricted. These factors ultimately limit a broader knowledge regarding the situation of CKD-MBD in Brazil. It is also difficult to assess the prevalence of the different types of ROD, which can harm the management and control of this important complication of CKD.

Within this context, the CKD-MBD outpatient clinic in the Hospital das Clínicas of the Universidade Federal de Pernambuco (HC-UFPE) has been operating for more than two decades, monitoring and treating patients on dialysis, kidney transplant recipients, or those still undergoing conservative CKD treatment who present with changes in mineral and bone metabolism.

Considering the clinical importance of CKD-MBD, the present study aims to describe the types of ROD and the clinical and osteometabolic profiles of dialysis patients undergoing bone biopsy in Pernambuco, with the aim of contributing to a better understanding, prevention and management of this complication within our setting and throughout Brazil.

## Methods

This was a cross-sectional, retrospective study, carried out at the CKD-MBD outpatient clinic at HC-UFPE. Patients on dialysis were selected from different dialysis clinics throughout the state of Pernambuco who were aged 18 years or over, had undergone a bone biopsy between January 2004 and September 2021 and whose bone fragment was suitable for a diagnosis of ROD. Patients with CKD receiving conservative management and kidney transplant recipients were excluded from the analysis. The present study was approved by the HC-UFPE Research Ethics Committee (CAAE: 60740322.0.0000.8807) and carried out in accordance with the principles of the Declaration of Helsinki. All patients signed an informed consent form in order to undergo a bone biopsy.

### Clinical, Demographic and Laboratory Parameters

The clinical, demographic, and laboratory parameters refer to the time of bone biopsy.

The following clinical and demographic data were analyzed: age, sex, time on dialysis, etiology of CKD, occurrence of fractures (pathological fractures reported by the patient and confirmed through physical examination and/or X-ray images, occurring during the period of dialysis were considered), previous use of at least one bottle of hydroxide aluminum during dialysis and submitted to parathyroidectomy.

The laboratory parameters analyzed were: total calcium (normal range - NR: 8.5−10.5 mg/dL), phosphorus (NR: 2.5−5.5 mg/dL), intact parathyroid hormone (iPTH; NR: 15−68.3 pg/mL) and total alkaline phosphatase (tALP). Total calcium and phosphorus were measured with colorimetric assays on the CMD 800i (Wiener Lab Group, Rosario, Argentina). iPTH was measured with a chemiluminescent immunoassay on the Architect i2000 SR (Abbott Park, Illinois, USA) and tALP with colorimetric assays on the CMD 800i (NR: 65−300 U/L. Wiener Lab Group, Rosario, Argentina), Architect i2000 SR (NR: 40−150 U/L. Abbott Park, Illinois, United States of America), or Beckman Coulter AU680 (NR: 35−104 U/L. Brea, United States of America). Due to changes in tALP reference values throughout the study period, we used the number of times that tALP (xtALP) was above the upper limit of normality.

### Bone Biopsy

A transiliac bone biopsy was performed following the double labeling protocol with tetracycline^6,7^. The main recommendations for performing bone biopsy were: to exclude aluminum toxicity, in the presence of persistent hypercalcemia and/or hyperphosphatemia or when laboratory tests were either incapable or insufficient to establish a diagnosis of ROD. The bone biopsy was performed at the HC-UFPE and the samples were processed and analyzed in a semiquantitative manner at the Renal Pathophysiology Laboratory-LIM 16 in the Medical School of Universidade de São Paulo (FMUSP). According to histological findings, patients were divided into two groups: high and low bone turnover. The high turnover group included patients diagnosed with OF and MD, while the low turnover group included those with ABD and OM^5^.

In addition, the presence of aluminum toxicity, iron toxicity, and osteoporosis was assessed. The diagnostic criterion for aluminum toxicity was 25% or more of the trabecular bone covered by metal, observed through the analysis of slides stained with solochrome azurine. This level was used for diagnosing aluminum toxicity during the initial years of the study period and was only later redefined to 30%. To assess the iron accumulation on the trabecular bone, Perls stain was used. Iron toxicity was defined as the presence of iron on 30% or more of the trabecular surface. Although the bone biopsy analysis was semiquantitative, the diagnosis of osteoporosis took into account low trabecular bone volume^8^ assessed in detail in all fields of the slide by a single experienced observer.

### Statistical Analysis

Since the numerical variables did not present a normal distribution, they are expressed as median and interquartile range. Categorical variables are presented as frequencies and percentages.

Comparative analyzes were carried out between the groups with high and low bone turnover, with or without aluminum toxicity, and with and without a diagnosis of osteoporosis using the non-parametric Wilcoxon test for numerical variables and the Chi-square and Fisher’s exact tests for categorical variables.

Statistical analyses were performed using R 3.6.3 (R Foundation for Statistical Computing, Vienna, Austria, 2019). A statistical significance level of 5% (p < 0.05) was considered.

## Results

Table 1 presents the clinical and laboratory characteristics of the patients. A total of 250 patients were studied, most were middle-aged, and had been on dialysis for a long period of time, 245 (98%) of whom were on hemodialysis. The main etiology of CKD was undetermined. The previous use of aluminum hydroxide was still common in the studied population. Information regarding the occurrence of fractures was available for 229 patients, 19 of whom (8.3%) had suffered at least one fracture. The main fracture sites were long bones (63.2%), hip (26.3%), and spine (10.5%). Parathyroidectomy surgery was performed on 64 patients, and in 19 of these, the surgical procedure occurred before bone biopsy.

**Table 1:** Demographic, clinical, and laboratory characteristics of the patients (n = 250)

**Age, in years**	48 (41 − 58)
**Female sex (N, %)**	144 (57.6%)
**Length of time on dialysis (months)**	108 (72 − 156)
**Etiology of CKD (N, %)**	
Indeterminate	100 (40%)
Arterial hypertension	57 (22.8%)
Others	44 (17.6%)
Chronic glomerulopathy	30 (12%)
Diabetes mellitus	19 (7.6%)
**Use of aluminum hydroxide^1^**	108 (47.1%)
**Fractures^1^**	19 (8.3%)
**Parathyroidectomy**	64 (25.6%)
Preliminary bone biopsy	19 (29.6%)
**Total calcium (mg/dL**)	9.8 (9.2 − 10.5)
**Phosphorus (mg/dL)**	5.8 (4.7 − 6.95)
**iPTH (pg/mL)**	1114 (238 − 1959)
**xtALP**	1.53 (0.86 − 3.62)

Abbreviations – CKD: chronic kidney disease; iPTH: Intact parathyroid hormone; xtALP: number of times that total alkaline phosphatase (U/L) was above the upper reference value for the method. Notes – Values are expressed as median and interquartile range (P25-P75) and frequency (percentage). ^1^n = 229.

With regard to the biochemical profile, 52% of the patients presented with hyperphosphatemia and, although the median total calcium was normal, we observed hypercalcemia in 24.2% of patients. The median iPTH was approximately 16 times above the upper limit of the method (1114 pg/mL). The vast majority of patients (77.4%) diagnosed with ABD presented iPTH levels equal to or less than twice the upper limit of the method, whereby the minimum and maximum iPTH levels in these patients were 5.8 and 307 pg/mL, respectively. The majority of patients had high tALP levels.

In relation to a diagnosis of ROD, OF was the most common type (54.4%), followed by MD (30.4%). ABD was present in 12.4% of the pati-ents and only 8 patients (3.2%) presented OM. Osteoporosis, aluminum toxicity, and iron toxicity were observed in 43.6%, 46.8%, and 27.5% of patients, respectively.

**Figure 1:**
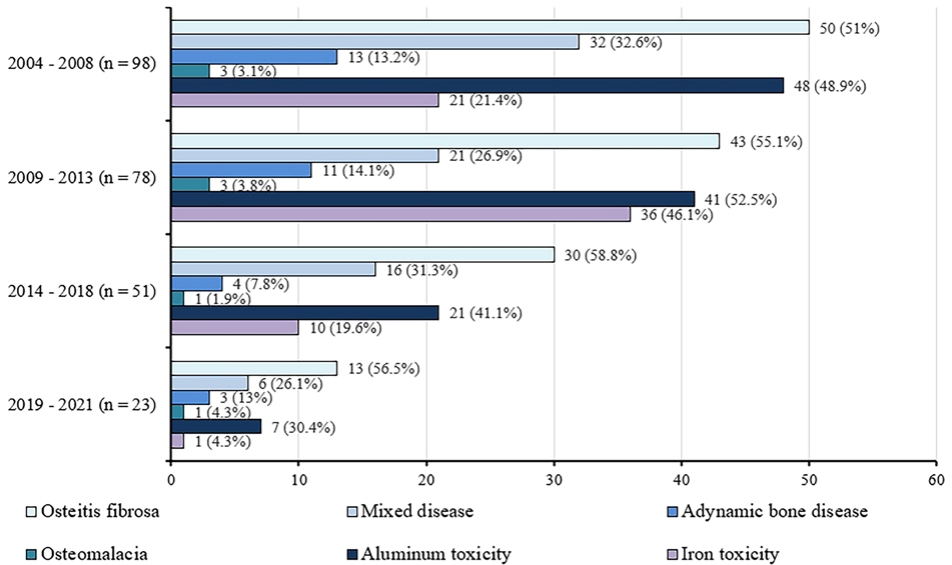
Distribution (n, %) of types of renal osteodystrophy and aluminum and iron toxicity over the study period.

**Figure 2:**
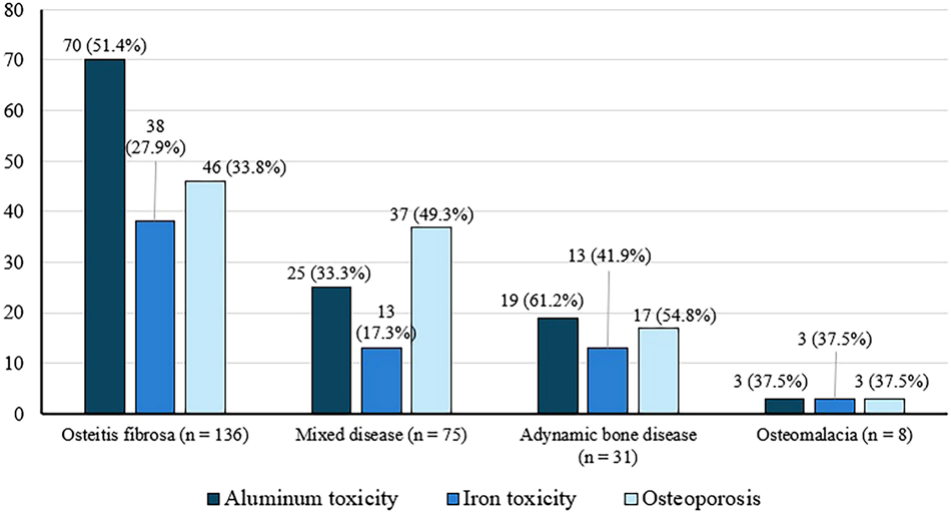
Distribution (n, %) of aluminum toxicity, iron toxicity, and osteoporosis in the different types of renal osteodystrophy.

Figure 1 presents the distribution of types of renal osteodystrophy and the frequency of aluminum and iron toxicity throughout the study period. Figure 2 shows the frequency of osteoporosis, aluminum toxicity, and iron toxicity in the different types of ROD.

Table 2 presents the comparative analysis of patients according to bone turnover. The occurrence of fractures (21 vs. 6.1%; p = 0.01) and iron toxicity (41 vs. 25%; p = 0.04) was more frequent in patients with low bone turnover and no difference was found in the prevalence of osteoporosis (p = 0.2). From a laboratory viewpoint, patients with high turnover presented higher levels of total calcium, phosphorus, iPTH, and tALP than patients with low turnover. There was no difference in the diagnosis of aluminum toxicity.

**Table 2:** Comparative analysis between groups according to bone turnover (n = 250)

Variables	High turnover N = 211	Low turnover N = 39		p^1^
**Age, in years**	48 (41 − 57)	47 (38 − 62)	0.9
**Female sex (N, %)**	125 (59.2%)	19 (48.7%)		0.2
**Etiology of CKD (N, %)**				0.8
Indeterminate	87 (41%)	13 (33%)		
Systemic arterial hypertension	46 (22%)	11 (28%)		
Others	36 (17%)	8 (21%)		
Chronic glomerulopathy	25 (12%)	5 (13%)		
Diabetes mellitus	17 (8.1%)	2 (5.1%)		
**Length of time on dialysis (months)**	108 (84 − 156)	96 (50 − 144)		0.061
**Use of aluminum hydroxide^2^**	93 (48%)	15 (44%)		0.7
**Fractures^2^**	12 (6.1%)	7 (21%)		0.01
**Calcium (mg/dL)**	9.9 (9.3 − 10.5)	9.28 (8.65 − 10.0)		0.006
**Phosphorus (mg/dL)**	5.9 (4.9 − 7.1)	4.95 (3.58 − 6.18)		<0.001
**iPTH (pg/mL)**	1395 (692 − 2125)	79 (30 − 129)		<0.001
**xtALP**	1.98 (1.0 − 4.2)	0.82 (0.50 − 1.22)		<0.001
**Osteoporosis**	88 (41.7%)	21 (53.8%)		0.2
**Aluminum toxicity**	95 (45%)	22 (56.4%)		0.2
**Iron toxicity**	52 (24.6%)	16 (41%)		0.04
**Total**	**211**	**39**		

Abbreviations – CKD: chronic kidney disease; iPTH: Intact parathyroid hormone; xtALP: number of times that total alkaline phosphatase (U/L) was above the upper reference value for the method. Notes – Values are expressed as median and interquartile range (P25-P75) and frequency (percentage). ^1^Wilcoxon test, Chi-square, or Fisher’s exact test; ^2^n = 229.

Table 3 presents the comparative analysis of patients according to the presence of aluminum toxicity. Those with aluminum intoxication had higher iPTH and alkaline phosphatase values, had fewer MD diagnoses, and were more associated with iron intoxication.

**Table 3:** Comparative analysis according to presence of aluminum toxicity (n = 250)

		Aluminum toxicity				
		Variables	Yes (N = 117)		No (N = 133)		p^1^
**Age, in years**	47 (39 – 59)		48 (41 – 56)		0.7
**Female sex (N, %)**	66 (56%)		55 (41%)		0.7
**Etiology of CKD (N, %)**			0.8
Indeterminate	42 (36%)		58 (44%)		
Systemic arterial hypertension	28 (24%)		29 (22%)		
Others	23 (20%)		21 (16%)		
Chronic glomerulopathy	14 (12%)		16 (12%)		
Diabetes mellitus	10 (8.5%)		9 (6.8%)		
**Length of time on dialysis (months)**	108 (71 – 168)		108 (71 – 156)		0.7
**Use of aluminum hydroxide^2^**	49 (46%)		59 (48%)		0.8
**Fractures^2^**	8 (7.6%)		11 (8.9%)		0.7
**Calcium (mg/dL)**	9.85 (9.25 – 10.4)		9.8 (9.1 – 10.6)		0.9
**Phosphorus (mg/dL)**	5.7 (4.6 – 9.6)		5.9 (4.7 – 6.96)		0.7
**iPTH (pg/mL)**	741 (130 – 1377)		1528 (614 – 2354)		<0.001
**xtALP**	1.21 (0.79 – 2.07)		2.77 (1.04 – 4.89)		<0.001
**OF**	70 (60%)		66 (50%)		0.11
**MD**	25 (21%)		51 (38%)		0.004
**ABD**	19 (16%)		12 (9%)		0.084
**OM**	3 (2.6%)		5 (3.8%)		0.7
**Osteoporosis**	51 (44%)		58 (44%)		0.9
**Iron toxicity**	57 (50%)		11 (8.3%)		<0.001
**Total**	**117**		**133**		

Abbreviations – CKD: chronic kidney disease; iPTH: Intact parathyroid hormone; xtALP: number of times that total alkaline phosphatase (U/L) was above the upper reference value for the method; OF: osteitis fibrosa; MD: mixed disease; ABD: adynamic bone disease; OM: osteomalacia. Notes – Values are expressed as median and interquartile range (P25-P75) and frequency (percentage). ^1^Wilcoxon test, Chi-square, or Fisher’s exact test. ^2^n = 229.

The comparative analysis of patients with and without osteoporosis is shown in Table 4. Those with osteoporosis were older, had more fractures, lower phosphorus levels, and fewer OF diagnoses.

**Table 4:** Comparative analysis according to the presence of osteoporosis (n = 250)

	Osteoporosis				
Variables	Yes (N = 109)		No (N = 141)		p^1^
**Age, in years**	51 (42 – 60)		47 (35 – 53)		0.034
**Female sex (N, %)**	64 (59%)		80 (57%)		0.8
**Etiology of CKD (N, %)**					0.9
Indeterminate	42 (39%)		58 (41%)		
Systemic arterial hypertension	24 (22%)		33 (23%)		
Others	22 (20%)		22 (16%)		
Chronic glomerulopathy	12 (11%)		18 (13%)		
Diabetes mellitus	9 (8.3%)		10 (7.1%)		
**Length of time on dialysis (months)**	120 (72 – 156)		108 (72 – 156)		0.8
**Use of aluminum hydroxide^2^**	41 (42%)		67 (51%)		0.2
**Fractures^2^**	13 (13%)		6 (4.6%)		0.021
**Calcium (mg/dL)**	9.7 (9.0 – 10.5)		9.8 (9.2 – 10.5)		0.6
**Phosphorus (mg/dL)**	5.4 (4.4 – 6.6)		6.0 (5.0 – 7.2)		0.014
**iPTH (pg/mL)**	1240 (207 – 1967)		1035 (252 – 1952)		0.8
**xtALP**	1.64 (0.84 – 4.36)		1.42 (0.87 – 3.13)		0.2
**OF**	50 (46%)		86 (61%)		0.017
**MD**	38 (35%)		38 (27%)		0.2
**ABD**	17 (16%)		14 (9.9%)		0.2
**OM**	4 (3.7%)		4 (2.8%)		0.7
**Aluminum toxicity**	51 (47%)		66 (47%)		0.9
**Iron toxicity**	27 (25%)		41 (29%)		0.4
**Total**	**109**		**141**		

Abbreviations – CKD: chronic kidney disease; iPTH: Intact parathyroid hormone; xtALP: number of times that total alkaline phosphatase (U/L) was above the upper reference value for the method; OF: osteitis fibrosa; MD: mixed disease; ABD: adynamic bone disease; OM: osteomalacia. Notes – Values are expressed as median and interquartile range (P25-P75) and frequency (percentage). ^1^Wilcoxon test, Chi-square, or Fisher’s exact test. ^2^n = 229.

## Discussion

Mineral and bone disease, by causing loss of bone mass, fractures, and vascular calcification, is a serious complication of CKD and contributes to poor clinical outcomes, especially those related to the cardiovascular system.

This study is the first to assess bone biopsies performed on dialysis patients in the state of Pernambuco, and is one of the few in the literature that has assessed CKD-MBD, through bone biopsy in a significant number of patients from the same center. We studied 250 patients followed at the CKD-MBD outpatient clinic at the HC-UFPE over a period of almost 20 years who had been referred by attending physicians from several dialysis clinics in the state of Pernambuco. The main reason for referral was secondary hyperparathyroidism that was difficult to control clinically, and in most cases, bone biopsy was performed to exclude aluminum toxicity, in the presence of persistent hypercalcemia and/or hyperphosphatemia or when laboratory tests were either incapable or insufficient to establish a diagnosis of ROD. Thus, our results may not reflect the true prevalence of the types of ROD in Pernambuco, as the study was carried out in a specialized outpatient clinic that obviously already admits more severe patients, and, therefore, contains a probable selection bias.

With regard to ROD diagnosis, high bone turnover prevailed during all periods assessed, thereby reflecting the fact that patients had been referred due to secondary hyperparathyroidism. Indeed, almost 85% of the patients presented with OF or MD, and low bone turnover was observed in around 15% of these, which was predominantly represented by ABD. Our results are very similar to those recently presented in a partial REBRABO^9^ analysis of biopsies from 2015 to 2018. In this study, the authors also found a high prevalence (75%) of high bone turnover and ABD in 16% of biopsies from 173 chronic kidney patients from all over Brazil^8^.

The ROD spectrum has undergone changes worldwide. These changes are due in part to the aging of dialysis patients, an increase in the number of people with diabetes, a better aluminum control, and chiefly, a more effective treatment of secondary hyperparathyroidism, in addition to the emergence of new drugs for treating CKD-BMD^10^. Thus, the high bone turnover that predominated in early studies has given way to low turnover^11,12,13,14^. Indeed, the prevalence of ABD is increasing and it is the most common type of ROD in some series^15,16,17,18,19^. In this context, Malluche et al.^16^, studying 630 bone biopsies from patients in the US and Europe carried out between 2003 and 2008 demonstrated that 58% and 24% of patients exhibited low and high bone turnover, respectively. Studies that included Brazilian patients both on dialysis and pre-dialysis also demonstrated a high prevalence of low bone turnover^15,17,20^. The most recent REBRABO analysis, now with data from 386 biopsy patients from 2015 to 2021, demonstrated that high bone turnover, although prevalent (63%), was already slightly lower than initially demonstrated, at the same time that the prevalence of ABD increased from 16% to 25%, thereby following the trend in developed countries^21^. Thus, the actual prevalence of different ROD types is not only a national challenge, but global one, since it involves many factors, including clinical and demographic differences in patients, such as age, race, underlying disease, and differences in public health treatment programs worldwide, which facilitate access to medications. All of these factors contribute to the non-standardization of ROD types.

As presented in Table 1, our patients were middle-aged adults who had been on dialysis for a long period of time, and although biopsies were performed during the 2000s, we nonetheless observed a high prevalence of aluminum toxicity on the trabecular bone. Indeed, 46.8% of our patients were diagnosed with aluminum toxicity. This prevalence was similar to the 38% aluminum toxicity observed throughout Brazil, as reported in the REBRABO study and confirmed in its most recent analysis^8,21^. The most recent analysis, when assessing data by geographic regions, demonstrated that 42% of the biopsies in the Northeastern region exhibited aluminum toxicity^21^. Brazilian data, however, contrasted with that of the rest of the world. Better control of water treatment for hemodialysis and the use of aluminum-free phosphorus chelators resulted in aluminum toxicity becoming increasingly less frequent in the US and Europe, reaching prevalences as low as 0.6%, as observed by Malluche et al.^16^.

Iron toxicity was found in 27.5% of our patients. Unlike aluminum, the effect of iron on bone metabolism remains uncertain^22^. We observed an association between iron toxicity and low bone turnover, a finding previously reported by other authors^23^.

Although the median age of our patients was less than 50 years, loss of bone mass was present, and more than 40% were suffering from osteoporosis, diagnosed by bone biopsy. Patients with osteoporosis were older and presented more fractures. Loss of bone mass is quite prevalent in CKD and increases the risk of fractures in this population. When compared to the general population, patients with CKD, especially those who have been on dialysis for a long period of time, have a significantly higher risk of fractures, which is associated with mortality^24^. Interestingly, although high turnover prevailed among our patients, those with osteoporosis were less frequently diagnosed with OF than those without osteoporosis. The prevalence of fractures was higher in patients with low bone turnover. Based on these findings, we were able to demonstrate that low bone turnover is no less important for bone mass loss and fracture risk in CKD, as other authors have observed^25,26,27^.

Since our study is a retrospective analysis of medical records of patients routinely monitored at a CKD-MBD outpatient clinic, some limitations need to be considered. These include the absence of a histomorphometric analysis of the bone biopsy and the lack of more accurate information concerning the amount of aluminum intake by patients. However, the main limitation is selection bias, since the patients studied represent a group of individuals that have been on dialysis for a long period, with more severe bone disease that is difficult to control. Therefore, our results may not reflect the true reality of ROD in the state of Pernambuco.

In summary, our study has revealed similar results to those observed throughout Brazil, confirming the high prevalence of high-turnover bone disease and also a prohibitively high prevalence of aluminum toxicity in bone. Being the first in our state, this study is of great importance, since it provides information that will certainly contribute to a better management of CKD-MBD in our region by revealing a photograph of the severity of bone disease in patients who are referred to our service. Furthermore, our results confirmed the important association between low bone turnover and the risk of fractures.

We hope that the information provided in this study can enrich knowledge about CKD-MBD in Pernambuco and Brazil, in order to mitigate the effects of CKD-BMD on the morbidity and mortality of dialysis patients.

## Data Availability

Data available upon request. The entire dataset supporting the findings of this study is available upon request to the corresponding author [Luiz Alberto Soares de Araújo Coutinho]. The dataset is not publicly available due to the fact that it contains information that compromises the privacy of the research participants.

## References

[1] Block GA, Klassen PS, Lazarus JM, Ofsthun N, Lowrie EG, Chertow GM. (2004). Mineral metabolism, mortality, and morbidity in maintenance hemodialysis.. J Am Soc Nephrol..

[2] Schlieper G, Hess K, Floege J, Marx N. (2016). The vulnerable patient with chronic kidney disease.. Nephrol Dial Transplant..

[3] Moody WE, Edwards NC, Chue CD, Ferro CJ, Townend JN. (2013). Arterial disease in chronic kidney disease.. Heart..

[4] Moe S, Drueke T, Cunningham J, Goodman W, Martin K, Olgaard K (2006). Definition, evaluation, and classification of renal osteodystrophy: a position statement from Kidney Disease: Improving Global Outcomes (KDIGO).. Kidney Int..

[5] Kidney Disease: Improving Global Outcomes (KDIGO) CKD-MBD Update Work Group. (2017). KDIGO clinical practice guideline for the diagnosis, evaluation, prevention, and treatment of chronic kidney disease-mineral and bone disorder (CKD-MBD).. Kidney Int Suppl..

[6] Malluche HH, Langub MC, Monier-Faugere MC. (1999). The role of bone biopsy in clinical practice and research.. Kidney Int Suppl..

[7] Barreto FC, Costa CRV, Reis LM, Custódio MR. (2018). Bone biopsy in nephrology practice.. J Bras Nefrol..

[8] Carbonara CEM, Reis LM, Quadros KRS, Roza NAV, Sano R, Carvalho AB (2020). Renal osteodystrophy and clinical outcomes: data from the Brazilian Registry of Bone Biopsies-REBRABO.. J Bras Nefrol..

[9] Oliveira RB, Barreto FC, Custódio MR, Gueiros JEB, Neves CL, Karohl C (2014). Brazilian Registry of Bone Biopsy (REBRABO): design, data, elements, and methodology.. J Bras Nefrol..

[10] Drüeke TB, Massy ZA. (2016). Changing bone patterns with progression of chronic kidney disease.. Kidney Int..

[11] Malluche HH, Ritz E, Lange HP, Kutschera L, Hodgson M, Seiffert U (1976). Bone histology in incipient and advanced renal failure.. Kidney Int..

[12] Hutchison AJ, Whitehouse RW, Boulton HF, Adams JE, Mawer EB, Freemont TJ (1993). Correlation of bone histology with parathyroid hormone, vitamin D3, and radiology in end-stage renal disease.. Kidney Int..

[13] Sherrard DJ, Hercz G, Pei Y, Maloney NA, Greenwood C, Manuel A (1993). The spectrum of bone disease in end-stage renal failure - an evolving disorder.. Kidney Int..

[14] Torres A, Lorenzo V, Hernández D, Rodríguez JC, Concepción MT, Rodríguez AP (1995). Bone disease in predialysis, hemodialysis, and CAPD patients: evidence of a better bone response to PTH.. Kidney Int..

[15] Barreto DV, Barreto FC, Carvalho AB, Cuppari L, Draibe AS, Dalboni MA (2008). Association of changes in bone remodeling and coronary calcification in hemodialysis patients: a prospective study.. Am J Kidney Dis..

[16] Malluche HH, Mawad HW, Monier-Faugere MC. (2011). Renal osteodystrophy in the first decade of the new millennium: analysis of 630 bone biopsies in black and white patients.. J Bone Miner Res..

[17] Sprague SM, Bellorin-Front E, Jorgetti V, Carvalho AB, Malluche HH, Ferreira A (2016). Diagnostic accuracy of bone turnover markers and bone histology in patients with CKD treated by dialysis.. Am J Kidney Dis..

[18] El-Husseini A, Abdalbary M, Lima F, Issa M, Ahmed M-T, Winkler M (2022). Low turnover renal osteodystrophy with abnormal bone quality and vascular calcification in patients with mild-to-moderate CKD.. Kidney Int Rep..

[19] Neto R, Pereira L, Magalhaes J, Quelhas-Santos J, Martins S, Carvalho C (2021). Sclerostin and DKK1 circulating levels associate with low bone turnover in patients with chronic kidney disease Stages 3 and 4.. Clin Kidney J..

[20] Barreto FC, Barreto DV, Canziani MEF, Tomiyama C, Higa A, Mozar A (2014). Association between indoxyl sulfate and bone histomorphometry in pre-dialysis chronic kidney disease patients.. J Bras Nefrol..

[21] Carbonara CEM, Roza NAV, Reis LM, Carvalho AB, Jorgetti V, Oliveira RB. (2023). Overview of renal osteodystrophy in Brazil: a cross-sectional study.. J Bras Nefrol..

[22] Custódio MR, Elias RM, Velasquez WD, Dos Reis LM, Oliveira IB, Moysés RMA (2018). The unexpected presence of iron in bone biopsies of hemodialysis patients.. Int Urol Nephrol..

[23] Van de Vyver FL, Visser WJ, D’Haese PC, De Broe ME. (1990). Iron overload and bone disease in chronic dialysis patients.. Nephrol Dial Transplant..

[24] Evenepoel P, Cunningham J, Ferrari S, Haarhaus M, Javaid MK, Lafage-Proust M-H (2021). European consensus statement on the diagnosis and management of osteoporosis in chronic kidney disease stages G4-G5D.. Nephrol Dial Transplant..

[25] Nagy E, Sobh MM, Abdalbary M, Elnagar S, Elrefaey R, Shabaka S (2022). Is adynamic bone always a disease? Lessons from patients with chronic kidney disease.. J Clin Med..

[26] Barreto FC, Barreto DV, Moyses RMA, Neves C, Jorgetti V, Draibe SA (2006). Osteoporosis in hemodialysis patients revisited by bone histomorphometry: a new insight into an old problem.. Kidney Int..

[27] Hughes-Austin JM, Katz R, Semba RD, Kritchevsky SB, Bauer DC, Sarnak MJ (2020). Biomarkers of bone turnover identify subsets of chronic kidney disease patients at higher risk for fracture.. J Clin Endocrinol Metab..

