# Supplementary material for: Renal osteodystrophy in a sample of patients on dialysis in Northeastern Brazil: a cross-sectional analysis
Source: J Bras Nefrol. 2025 Apr 14;47(2):e20240174. doi: 10.1590/2175-8239-JBN-2024-0174en (PMC12147466; doi:10.1590/2175-8239-JBN-2024-0174en)
Supplement: Translation

# Translation: Osteodistrofia renal em uma amostra de pacientes em diálise no Nordeste do Brasil: uma análise transversal

**DOI:** 10.1590/2175-8239-JBN-2024-0174pt

**Authors:** Luiz Alberto Soares de Araújo Coutinho, Ana Paula Santana Gueiros, Júlia Braga Vaz, Eduarda Cerqueira Russo, Vanda Jorgetti, José Edevanilson de Barros Gueiros

**Affiliations:** 1 Universidade Federal de Pernambuco, Empresa Brasileira de Serviços Hospitalares, Hospital das Clínicas, Serviço de Nefrologia, Recife, PE, Brazil.; 2 Universidade de São Paulo, Faculdade de Medicina, Laboratório de Investigação Médica, São Paulo, SP, Brazil.

**Keywords:** Insuficiência Renal Crônica, Diálise, Distúrbio Mineral e Ósseo na Doença Renal Crônica, Biópsia Óssea

## Abstract

**Introdução:**

Os distúrbios do metabolismo mineral e ósseo na doença renal crônica (DMO-DRC) constituem uma das complicações mais significativas da DRC. O objetivo deste estudo foi descrever os tipos de osteodistrofia renal (OR) e os perfis clínico e osteometabólico de pacientes em diálise submetidos à biópsia óssea em Pernambuco.

**Métodos:**

Foi realizado um estudo transversal e retrospectivo que avaliou pacientes submetidos à biópsia entre janeiro de 2004 e setembro de 2021. Foram excluídos pacientes em tratamento conservador para DRC, receptores de transplante renal e aqueles com biópsias inadequadas. Todos os parâmetros clínicos e laboratoriais foram obtidos no momento da biópsia.

**Resultados:**

Foram avaliados 250 pacientes com idade mediana de 48 anos (57,6% mulheres) e em diálise por um período mediano de 108 meses. Com relação ao diagnóstico de OR, a prevalência foi: osteíte fibrosa (54,5%), doença mista (30,4%), doença óssea adinâmica (12,4%) e osteomalácia (3,2%). As prevalências de osteoporose, toxicidade por alumínio e toxicidade por ferro foram de 43,6%, 46,8% e 27,5%, respectivamente.

**Conclusão:**

Demonstramos uma alta prevalência de doenças relacionadas à elevada remodelação óssea e à toxicidade do alumínio.

## Introdução

Os distúrbios do metabolismo mineral e ósseo na doença renal crônica (DMO-DRC) são uma das complicações mais significativas da DRC^1^. Como consequência da DMO-DRC, os pacientes em diálise sofrem perda óssea e apresentam um risco aumentado de fraturas, além de apresentarem calcificação vascular precoce e acelerada. Essas manifestações se somam e contribuem para desfechos clínicos desfavoráveis e elevada mortalidade cardiovascular nesses pacientes^1,2,3^.

O termo osteodistrofia renal (OR) é utilizado para descrever as alterações histológicas avaliadas por meio de análises semiquantitativas e histomorfométricas da biópsia óssea, empregando o sistema RMV (remodelação, mineralização e volume). A OR é, portanto, um dos componentes da DMO-DRC e pode ser classificada como alta ou baixa remodelação óssea. A alta remodelação compreende os diagnósticos de osteíte fibrosa (OF) e doença mista (DM), enquanto a baixa remodelação é representada pela doença óssea adinâmica (DOA) e osteomalácia (OM). A toxicidade por alumínio, em diferentes graus, pode estar presente em qualquer tipo de OR^4,5^.

No Brasil, existem poucos centros especializados no tratamento da DMO-DRC. A realização de procedimentos de biópsia óssea e, mais especificamente, o processamento e a análise diagnóstica das amostras são ainda mais restritos. Esses fatores acabam limitando um conhecimento mais amplo sobre a situação da DMO-DRC no Brasil. Também é difícil avaliar a prevalência dos diferentes tipos de OR, o que pode prejudicar o manejo e o controle dessa importante complicação da DRC.

Nesse contexto, o ambulatório de DMO-DRC do Hospital das Clínicas da Universidade Federal de Pernambuco (HC-UFPE) atua há mais de duas décadas, monitorando e tratando pacientes em diálise, receptores de transplante renal ou aqueles ainda em tratamento conservador da DRC que apresentam alterações no metabolismo mineral e ósseo.

Considerando a importância clínica da DMO-DRC, o presente estudo tem como objetivo descrever os tipos de OR e os perfis clínico e osteometabólico de pacientes em diálise submetidos à biópsia óssea em Pernambuco, com o intuito de contribuir para um melhor entendimento, prevenção e manejo dessa complicação em nosso contexto e em todo o Brasil.

## Métodos

Este foi um estudo transversal e retrospectivo, realizado no ambulatório de DMO-DRC do HC-UFPE. Foram selecionados pacientes em diálise de diferentes clínicas de diálise em todo o estado de Pernambuco, com idade igual ou superior a 18 anos, submetidos a uma biópsia óssea entre janeiro de 2004 e setembro de 2021 e cujo fragmento ósseo era adequado para o diagnóstico de OR. Pacientes com DRC sob manejo conservador e receptores de transplante renal foram excluídos da análise. O presente estudo foi aprovado pelo Comitê de Ética em Pesquisa do HC-UFPE (CAAE: 60740322.0.0000.8807) e realizado de acordo com os princípios da Declaração de Helsinque. Todos os pacientes assinaram um termo de consentimento livre e esclarecido para a realização da biópsia óssea.

### Parâmetros Clínicos, Demográficos e Laboratoriais

Os parâmetros clínicos, demográficos e laboratoriais referem-se ao momento da biópsia óssea.

Foram analisados os seguintes dados clínicos e demográficos: idade, sexo, tempo de diálise, etiologia da DRC, ocorrência de fraturas (foram consideradas as fraturas patológicas relatadas pelo paciente e confirmadas por meio de exame físico e/ou imagens de raios X, ocorridas durante o período de diálise), uso prévio de pelo menos um frasco de hidróxido de alumínio durante a diálise e realização de paratireoidectomia.

Os parâmetros laboratoriais analisados foram: cálcio total (intervalo normal - IN: 8,5–10,5 mg/dL), fósforo (IN: 2,5–5,5 mg/dL), paratormônio intacto (iPTH; IN: 15–68,3 pg/mL) e fosfatase alcalina total (FAt). O cálcio total e o fósforo foram medidos por ensaios colorimétricos no CMD 800i (*Wiener Lab Group*, Rosário, Argentina). O iPTH foi medido por meio de imunoensaio quimioluminescente no Architect i2000 SR (*Abbott Park*, Illinois, EUA) e a FAt por ensaios colorimétricos no CMD 800i (IN: 65–300 U/L. *Wiener Lab Group*, Rosário, Argentina), Architect i2000 SR (IN: 40–150 U/L. *Abbott Park*, Illinois, EUA) ou Beckman Coulter AU680 (IN: 35–104 U/L. Brea, EUA). Devido a alterações nos valores de referência da FAt durante o período do estudo, utilizamos o número de vezes que a FAt (xFAt) esteve acima do limite superior da normalidade.

### Biopsia Óssea

Foi realizada uma biópsia óssea transilíaca seguindo o protocolo de dupla marcação com tetraciclina^6,7^. As principais recomendações para a realização da biópsia óssea foram: excluir a toxicidade por alumínio, na presença de hipercalcemia e/ou hiperfosfatemia persistentes ou quando os exames laboratoriais fossem incapazes ou insuficientes para estabelecer o diagnóstico de OR. A biópsia óssea foi realizada no HC-UFPE e as amostras foram processadas e analisadas de forma semiquantitativa no Laboratório de Fisiopatologia Renal-LIM 16 da Faculdade de Medicina da Universidade de São Paulo (FMUSP). De acordo com os achados histológicos, os pacientes foram divididos em dois grupos: alta e baixa remodelação óssea. O grupo de alta remodelação incluiu pacientes diagnosticados com OF e DM, enquanto o grupo de baixa remodelação incluiu aqueles com DOA e OM^5^.

Além disso, avaliou-se a presença de toxicidade por alumínio, toxicidade por ferro e osteoporose. O critério diagnóstico para toxicidade por alumínio foi 25% ou mais do osso trabecular coberto por metal, observado por meio da análise de lâminas coradas com solocromo azurina. Esse nível foi utilizado para o diagnóstico de toxicidade por alumínio nos anos iniciais do período de estudo e somente mais tarde foi redefinido para 30%. Para avaliar o acúmulo de ferro no osso trabecular, foi utilizada a coloração de Perls. A toxicidade por ferro foi definida como a presença de ferro em 30% ou mais da superfície trabecular. Embora a análise da biópsia óssea tenha sido semiquantitativa, o diagnóstico de osteoporose considerou o baixo volume ósseo trabecular^8^ avaliado detalhadamente em todos os campos da lâmina por um único observador experiente.

### Análise Estatística

Como as variáveis numéricas não apresentaram distribuição normal, elas foram expressas como mediana e intervalo interquartil. As variáveis categóricas foram apresentadas como frequências e porcentagens.

Foram realizadas análises comparativas entre os grupos com alta e baixa remodelação óssea, com ou sem toxicidade por alumínio e com e sem diagnóstico de osteoporose, utilizando o teste não paramétrico de Wilcoxon para variáveis numéricas e os testes qui-quadrado e exato de Fisher para variáveis categóricas.

As análises estatísticas foram realizadas com o software R 3.6.3 (*R Foundation for Statistical Computing*, Viena, Áustria, 2019). Foi considerado um nível de significância estatística de 5% (p < 0,05).

## Resultados

A Tabela 1 apresenta as características clínicas e laboratoriais dos pacientes. Foram estudados 250 pacientes, sendo a maioria de meia-idade e submetidos à diálise por um longo período, dos quais 245 (98%) estavam em hemodiálise. A principal etiologia da DRC não foi determinada. O uso prévio de hidróxido de alumínio ainda era comum na população estudada. Informações sobre a ocorrência de fraturas estavam disponíveis para 229 pacientes, dos quais 19 (8,3%) haviam sofrido pelo menos uma fratura. Os principais locais de fratura foram ossos longos (63,2%), quadril (26,3%) e coluna vertebral (10,5%). A cirurgia de paratireoidectomia foi realizada em 64 pacientes e, em 19 deles, o procedimento cirúrgico ocorreu antes da biópsia óssea.

**Tabela 1:** Características demográficas, clínicas e laboratoriais dos pacientes (n = 250)

**Idade, em anos**	48 (41 – 58)
**Sexo feminino (N, %)**	144 (57,6%)
**Duração da diálise (meses)**	108 (72 – 156)
**Etiologia da DRC (N, %)**	
Indeterminada	100 (40%)
Hipertensão arterial	57 (22,8%)
Outras	44 (17,6%)
Glomerulopatia crônica	30 (12%)
Diabetes *mellitus*	19 (7,6%)
**Uso de hidróxido de alumínio^1^**	108 (47,1%)
**Fraturas^1^**	19 (8,3%)
**Paratireoidectomia**	64 (25,6%)
Biópsia óssea preliminar	19 (29,6%)
**Cálcio total (mg/dL)**	9,8 (9,2 – 10,5)
**Fósforo (mg/dL)**	5,8 (4,7 – 6,95)
**iPTH (pg/mL)**	1114 (238 – 1959)
**xFAt**	1,53 (0,86 – 3,62)

Abreviaturas – DRC: doença renal crônica; iPTH: Paratormônio intacto; xFAt: número de vezes em que a fosfatase alcalina total (U/L) esteve acima do valor de referência superior para o método. Notas – Os valores foram expressos como mediana e intervalo interquartil (P25-P75) e frequência (porcentagem). ^1^n = 229.

Com relação ao perfil bioquímico, 52% dos pacientes apresentaram hiperfosfatemia e, embora a mediana do cálcio total tenha sido normal, observou-se hipercalcemia em 24,2% dos pacientes. A mediana do iPTH ficou aproximadamente 16 vezes acima do limite superior do método (1114 pg/mL). A grande maioria dos pacientes (77,4%) diagnosticados com DOA apresentou níveis de iPTH iguais ou inferiores ao dobro do limite superior do método, de modo que os níveis mínimo e máximo de iPTH nesses pacientes foram de 5,8 e 307 pg/mL, respectivamente. A maioria dos pacientes apresentou níveis elevados de FAt.

Quanto ao diagnóstico de OR, OF foi a forma mais comum (54,4%), seguida por DM (30,4%). A DOA esteve presente em 12,4% dos pacientes e apenas 8 (3,2%) apresentaram OM. Osteoporose, toxicidade por alumínio e toxicidade por ferro foram observadas em 43,6%, 46,8% e 27,5% dos pacientes, respectivamente.

A Figura 1 apresenta a distribuição dos tipos de osteodistrofia renal e a frequência de toxicidade por alumínio e ferro ao longo do período do estudo. A Figura 2 mostra a frequência de osteoporose, toxicidade por alumínio e toxicidade por ferro nos diferentes tipos de OR.

**Figura 1:** Distribuição (n, %) dos tipos de osteodistrofia renal e toxicidade por alumínio e ferro durante o período do estudo.

**Figura 2:** Distribuição (n, %) de toxicidade por alumínio, toxicidade por ferro e osteoporose nos diferentes tipos de osteodistrofia renal.

A Tabela 2 apresenta a análise comparativa dos pacientes de acordo com a remodelação óssea. A ocorrência de fraturas (21 vs. 6,1%; p = 0,01) e toxicidade por ferro (41 vs. 25%; p = 0,04) foi mais frequente em pacientes com baixa remodelação óssea e não foi encontrada diferença na prevalência de osteoporose (p = 0,2). Do ponto de vista laboratorial, pacientes com alta remodelação apresentaram níveis mais elevados de cálcio total, fósforo, iPTH e FAt do que pacientes com baixa remodelação. Não houve diferença no diagnóstico de toxicidade por alumínio.

**Tabela 2:** Análise comparativa entre grupos de acordo com a remodelação óssea (n = 250)

Variáveis	Alta remodelação N = 211	Baixa remodelação N = 39	p^1^
**Idade, em anos**	48 (41 – 57)	47 (38 – 62)	0,9
**Sexo feminino (N, %)**	125 (59,2%)	19 (48,7%)	0,2
**Etiologia da DRC (N, %)**					0,8
**Indeterminada**	87 (41%)	13 (33%)	
**Hipertensão arterial sistêmica**	46 (22%)	11 (28%)	
**Outras**	36 (17%)	8 (21%)	
**Glomerulopatia crônica**	25 (12%)	5 (13%)	
**Diabetes *mellitus***	17 (8,1%)	2 (5,1%)	
**Duração da diálise (meses)**	108 (84 – 156)	96 (50 – 144)	0,061
**Uso de hidróxido de alumínio^2^**	93 (48%)	15 (44%)	0,7
**Fraturas^2^**	12 (6,1%)	7 (21%)	0,01
**Cálcio (mg/dL)**	9,9 (9,3 – 10,5)	9,28 (8,65 – 10,0)	0,006
**Fósforo (mg/dL)**	5,9 (4,9 – 7,1)	4,95 (3,58 – 6,18)	<0,001
**iPTH (pg/mL)**	1395 (692 – 2125)	79 (30 – 129)	<0,001
**xFAt**	1,98 (1,0 – 4,2)	0,82 (0,50 – 1,22)	<0,001
**Osteoporose**	88 (41,7%)	21 (53,8%)	0,2
**Toxicidade por alumínio**	95 (45%)	22 (56,4%)	0,2
**Toxicidade por ferro**	52 (24,6%)	16 (41%)	0,04
**Total**	**211**	**39**	

Abreviaturas – DRC: doença renal crônica; iPTH: Paratormônio intacto; xFAt: número de vezes em que a fosfatase alcalina total (U/L) esteve acima do valor de referência superior para o método. Notas – Os valores foram expressos como mediana e intervalo interquartil (P25-P75) e frequência (porcentagem). ^1^Teste de Wilcoxon, qui-quadrado ou teste exato de Fisher; ^2^n = 229.

A Tabela 3 apresenta a análise comparativa dos pacientes de acordo com a presença de toxicidade por alumínio. Aqueles com intoxicação por alumínio apresentaram valores mais elevados de iPTH e fosfatase alcalina, tiveram menos diagnósticos de DM e foram mais associados à intoxicação por ferro.

**Tabela 3:** Análise comparativa de acordo com a presença de toxicidade por alumínio (n = 250)

	Toxicidade por alumínio		
Variáveis	Sim (N = 117)	Não (N = 133)	p^1^
**Idade, em anos**	47 (39 – 59)	48 (41 – 56)	0,7
**Sexo feminino (N, %)**	66 (56%)	55 (41%)	0,7
**Etiologia da DRC (N, %)**					0,8
Indeterminada	42 (36%)	58 (44%)	
Hipertensão arterial sistêmica	28 (24%)	29 (22%)	
Outras	23 (20%)	21 (16%)	
Glomerulopatia crônica	14 (12%)	16 (12%)	
Diabetes *mellitus*	10 (8,5%)	9 (6,8%)	
**Duração da diálise (meses)**	108 (71 – 168)	108 (71 – 156)	0,7
**Uso de hidróxido de alumínio^2^**	49 (46%)	59 (48%)	0,8
**Fraturas^2^**	8 (7,6%)	11 (8,9%)	0,7
**Cálcio (mg/dL)**	9,85 (9,25 – 10,4)	9,8 (9,1 – 10,6)	0,9
**Fósforo (mg/dL)**	5,7 (4,6 – 9,6)	5,9 (4,7 – 6,96)	0,7
**iPTH (pg/mL)**	741 (130 – 1377)	1528 (614 – 2354)	<0,001
**xFAt**	1,21 (0,79 – 2,07)	2,77 (1,04 – 4,89)	<0,001
**OF**	70 (60%)	66 (50%)	0,11
**DM**	25 (21%)	51 (38%)	0,004
**DOA**	19 (16%)	12 (9%)	0,084
**OM**	3 (2,6%)	5 (3,8%)	0,7
**Osteoporose**	51 (44%)	58 (44%)	0,9
**Toxicidade por ferro**	57 (50%)	11 (8,3%)	<0,001
**Total**	**117**	**133**	

Abreviaturas – DRC: doença renal crônica; iPTH: Paratormônio intacto; xFAt: número de vezes em que a fosfatase alcalina total (U/L) esteve acima do valor de referência superior para o método; OF: osteíte fibrosa; DM: doença mista; DOA: doença óssea adinâmica; OM: osteomalácia. Notas – Os valores foram expressos como mediana e intervalo interquartil (P25-P75) e frequência (porcentagem). ^1^Teste de Wilcoxon, qui-quadrado ou teste exato de Fisher. ^2^n = 229.

A análise comparativa de pacientes com e sem osteoporose foi demonstrada na Tabela 4. Os pacientes com osteoporose eram mais velhos, apresentaram mais fraturas, níveis mais baixos de fósforo e menos diagnósticos de OF.

**Tabela 4:** Análise comparativa de acordo com a presença de osteoporose (n = 250)

	Osteoporose		
Variáveis	Sim (N = 109)	Não (N = 141)	p^1^
**Idade, em anos**	51 (42 – 60)	47 (35 – 53)	0,034
**Sexo feminino (N, %)**	64 (59%)	80 (57%)	0,8
**Etiologia da DRC (N, %)**			0,9
Indeterminada	42 (39%)	58 (41%)	
Hipertensão arterial sistêmica	24 (22%)	33 (23%)	
Outras	22 (20%)	22 (16%)	
Glomerulopatia crônica	12 (11%)	18 (13%)	
Diabetes *mellitus*	9 (8,3%)	10 (7,1%)	
**Duração da diálise (meses)**	120 (72 – 156)	108 (72 – 156)	0,8
**Uso de hidróxido de alumínio^2^**	41 (42%)	67 (51%)	0,2
**Fraturas^2^**	13 (13%)	6 (4,6%)	0,021
**Cálcio (mg/dL)**	9,7 (9,0 – 10,5)	9,8 (9,2 – 10,5)	0,6
**Fósforo (mg/dL)**	5,4 (4,4 – 6,6)	6,0 (5,0 – 7,2)	0,014
**iPTH (pg/mL)**	1240 (207 – 1967)	1035 (252 – 1952)	0,8
**xFAt**	1,64 (0,84 – 4,36)	1,42 (0,87 – 3,13)	0,2
**OF**	50 (46%)	86 (61%)	0,017
**DM**	38 (35%)	38 (27%)	0,2
**DOA**	17 (16%)	14 (9,9%)	0,2
**OM**	4 (3,7%)	4 (2,8%)	0,7
**Toxicidade por alumínio**	51 (47%)	66 (47%)	0,9
**Toxicidade por ferro**	27 (25%)	41 (29%)	0,4
**Total**	**109**	**141**	

Abreviaturas – DRC: doença renal crônica; iPTH: Paratormônio intacto; xFAt: número de vezes em que a fosfatase alcalina total (U/L) esteve acima do valor de referência superior para o método; OF: osteíte fibrosa; DM: doença mista; DOA: doença óssea adinâmica; OM: osteomalácia. Notas – Os valores foram expressos como mediana e intervalo interquartil (P25-P75) e frequência (porcentagem). ^1^Teste de Wilcoxon, qui-quadrado ou teste exato de Fisher. ^2^n = 229.

## Discussão

A doença mineral óssea, ao causar perda de massa óssea, fraturas e calcificação vascular, é uma complicação grave da DRC e contribui para desfechos clínicos desfavoráveis, especialmente aqueles relacionados ao sistema cardiovascular.

Este estudo é o primeiro a avaliar biópsias ósseas realizadas em pacientes em diálise no estado de Pernambuco e é um dos poucos na literatura que investigou a DMO-DRC, por meio de biópsia óssea, em um número significativo de pacientes de um mesmo centro. Estudamos 250 pacientes acompanhados no ambulatório de DMO-DRC do HC-UFPE por um período de quase 20 anos, encaminhados por médicos assistentes de diversas clínicas de diálise do estado de Pernambuco. O principal motivo para o encaminhamento foi o hiperparatireoidismo secundário de difícil controle clínico e, na maioria dos casos, a biópsia óssea foi realizada para excluir a toxicidade por alumínio, na presença de hipercalcemia e/ou hiperfosfatemia persistentes ou quando os exames laboratoriais fossem incapazes ou insuficientes para estabelecer o diagnóstico de OR. Dessa forma, nossos resultados podem não refletir a real prevalência dos tipos de OR em Pernambuco, uma vez que o estudo foi realizado em um ambulatório especializado que evidentemente já recebe pacientes mais graves e, portanto, apresenta um provável viés de seleção.

Com relação ao diagnóstico de OR, a alta remodelação óssea prevaleceu em todos os períodos avaliados, refletindo assim o fato de que os pacientes haviam sido encaminhados devido ao hiperparatireoidismo secundário. De fato, quase 85% dos pacientes apresentaram OF ou DM, enquanto a baixa remodelação óssea foi observada em aproximadamente 15% dos casos, representada predominantemente por DOA. Nossos resultados são muito semelhantes aos apresentados recentemente em uma análise parcial do REBRABO^9^ referente a biópsias realizadas entre 2015 e 2018. Nesse estudo, os autores também encontraram uma prevalência elevada (75%) de alta remodelação óssea e DOA em 16% das biópsias de 173 pacientes com doença renal crônica de todo o Brasil^8^.

O espectro da OR passou por alterações em nível mundial. Essas mudanças se devem, em parte, ao envelhecimento dos pacientes em diálise, ao aumento do número de pessoas com diabetes, a um melhor controle do alumínio e, principalmente, a um tratamento mais eficaz do hiperparatireoidismo secundário, além do surgimento de novos medicamentos para o tratamento da DMO-DRC^10^. Assim, a alta remodelação óssea, que predominou nos primeiros estudos, tem dado lugar à baixa remodelação^11,12,13,14^. Com efeito, a prevalência de DOA está aumentando e, em algumas séries, é o tipo mais comum de OR^15,16,17,18,19^. Nesse contexto, Malluche et al.^16^, ao estudarem 630 biópsias ósseas de pacientes nos EUA e na Europa, realizadas entre 2003 e 2008, demonstraram que 58% e 24% dos pacientes apresentaram baixa e alta remodelação óssea, respectivamente. Estudos que incluíram pacientes brasileiros em diálise e pré-diálise também demonstraram uma elevada prevalência de baixa remodelação óssea^15,17,20^. A mais recente análise do REBRABO, agora com dados de 386 pacientes com biópsias realizadas de 2015 a 2021, demonstrou que a alta remodelação óssea, embora prevalente (63%), já estava um pouco abaixo do inicialmente demonstrado, ao mesmo tempo em que a prevalência de DOA aumentou de 16% para 25%, seguindo assim a tendência dos países desenvolvidos^21^. Assim, a prevalência real dos diferentes tipos de OR não é apenas um desafio nacional, mas global, pois envolve diversos fatores que incluem diferenças clínicas e demográficas dos pacientes como idade, raça, doença subjacente e diferenças nos programas de tratamento de saúde pública em todo o mundo, que facilitam o acesso a medicamentos. Todos esses fatores contribuem para a não padronização dos tipos de OR.

Conforme apresentado na Tabela 1, nossos pacientes eram adultos de meia-idade que estiveram em diálise por um longo período e, embora as biópsias tenham sido realizadas durante a década de 2000, ainda assim observamos uma alta prevalência de toxicidade por alumínio no osso trabecular. De fato, 46,8% de nossos pacientes foram diagnosticados com toxicidade por alumínio. Essa prevalência foi semelhante aos 38% de toxicidade por alumínio observados em todo o Brasil, conforme relatado no estudo REBRABO e confirmado em sua análise mais recente^8,21^. A análise mais recente, ao avaliar os dados por regiões geográficas, demonstrou que 42% das biópsias na região Nordeste apresentaram toxicidade por alumínio^21^. Os dados brasileiros, entretanto, contrastam com os do restante do mundo. Um melhor controle do tratamento da água para hemodiálise e o uso de quelantes de fósforo livres de alumínio resultaram em uma toxicidade por alumínio cada vez menos frequente nos EUA e na Europa, atingindo prevalências tão baixas quanto 0,6%, conforme observado por Malluche et al.^16^.

A toxicidade por ferro foi encontrada em 27,5% dos nossos pacientes. Diferentemente do alumínio, o efeito do ferro no metabolismo ósseo permanece incerto^22^. Observamos uma associação entre a toxicidade por ferro e a baixa remodelação óssea, um achado relatado anteriormente por outros autores^23^.

Embora a idade mediana de nossos pacientes fosse inferior a 50 anos, a perda de massa óssea esteve presente, e mais de 40% sofriam de osteoporose, diagnosticada por biópsia óssea. Os pacientes com osteoporose eram mais velhos e apresentaram mais fraturas. A perda de massa óssea é bastante prevalente na DRC e aumenta o risco de fraturas nessa população. Quando comparados à população em geral, pacientes com DRC, especialmente aqueles em diálise por um longo período, apresentam um risco significativamente maior de fraturas, o que está associado à mortalidade^24^. Curiosamente, embora a alta remodelação tenha prevalecido entre nossos pacientes, aqueles com osteoporose foram menos frequentemente diagnosticados com OF do que aqueles sem osteoporose. A prevalência de fraturas foi maior em pacientes com baixa remodelação óssea. Com base nesses achados, pudemos demonstrar que a baixa remodelação óssea não é menos importante para a perda de massa óssea e o risco de fraturas na DRC, como observaram outros autores^25,26,27^.

Como nosso estudo é uma análise retrospectiva de registros médicos de pacientes monitorados rotineiramente em um ambulatório de DMO-DRC, algumas limitações precisam ser consideradas. Tais limitações incluem a ausência de uma análise histomorfométrica da biópsia óssea e a falta de informações mais precisas sobre a quantidade de ingestão de alumínio pelos pacientes. No entanto, a principal limitação é o viés de seleção, uma vez que os pacientes estudados representam um grupo de indivíduos submetidos à diálise por um longo período, com doença óssea mais grave e de difícil controle. Portanto, nossos resultados podem não refletir a verdadeira realidade da OR no estado de Pernambuco.

Em resumo, nosso estudo revelou resultados semelhantes aos observados em todo o Brasil, confirmando a prevalência elevada de doença óssea de alta remodelação e uma prevalência proibitivamente alta de toxicidade por alumínio nos ossos. Por ser o primeiro em nosso estado, este estudo é de grande importância, uma vez que fornece informações que certamente contribuirão para um melhor manejo da DMO-DRC em nossa região, ao revelar um retrato da gravidade da doença óssea em pacientes encaminhados ao nosso serviço. Além disso, nossos resultados confirmaram a importante associação entre a baixa remodelação óssea e o risco de fraturas.

Esperamos que as informações fornecidas neste estudo possam enriquecer o conhecimento sobre DMO-DRC em Pernambuco e no Brasil, a fim de mitigar os efeitos da doença na morbidade e mortalidade dos pacientes em diálise.

## Data Availability

Dados disponíveis mediante solicitação. Todo o conjunto de dados que dá suporte aos resultados deste estudo está disponível mediante solicitação ao autor correspondente [Luiz Alberto Soares de Araújo Coutinho]. O conjunto de dados não está publicamente disponível devido a por conter informações que comprometem a privacidade dos participantes da pesquisa.
